# Hypoxia-mediated programmed cell death is involved in the formation of wooden breast in broilers

**DOI:** 10.1186/s40104-024-01036-1

**Published:** 2024-06-06

**Authors:** Xinrui Zhang, Tong Xing, Lin Zhang, Liang Zhao, Feng Gao

**Affiliations:** College of Animal Science and Technology, Key Laboratory of Animal Origin Food Production and Safety Guarantee of Jiangsu Province, Jiangsu Collaborative Innovation Center of Meat Production and Processing, Quality and Safety Control, Nanjing Agricultural University, No. 1 Weigang, Nanjing, Jiangsu 210095 P.R. China

**Keywords:** Apoptosis, Autophagy, Broiler chicken, Hypoxia, Necroptosis, Wooden breast

## Abstract

**Background:**

Wooden breast (WB) myopathy is a common myopathy found in commercial broiler chickens worldwide. Histological examination has revealed that WB myopathy is accompanied by damage to the pectoralis major (PM) muscle. However, the underlying mechanisms responsible for the formation of WB in broilers have not been fully elucidated. This study aimed to investigate the potential role of hypoxia-mediated programmed cell death (PCD) in the formation of WB myopathy.

**Results:**

Histological examination and biochemical analysis were performed on the PM muscle of the control (CON) and WB groups. A significantly increased thickness of the breast muscle in the top, middle, and bottom portions (*P*<0.01) was found along with pathological structure damage of myofibers in the WB group. The number of capillaries per fiber in PM muscle, and the levels of pO_2_ and sO_2_ in the blood, were significantly decreased (*P* < 0.01), while the levels of pCO_2_ and TCO_2_ in the blood were significantly increased (*P* < 0.05), suggesting hypoxic conditions in the PM muscle of the WB group. We further evaluated the PCD-related pathways including autophagy, apoptosis, and necroptosis to understand the consequence response to enhanced hypoxic conditions in the PM muscle of birds with WB. The ratio of LC3 II to LC3 I, and the autophagy-related factors HIF-1α, BNIP3, Beclin1, AMPKα, and ULK1 at the mRNA and protein levels, were all significantly upregulated (*P* < 0.05), showing that autophagy occurred in the PM muscle of the WB group. The apoptotic index, as well as the expressions of Bax, Cytc, caspase 9, and caspase 3, were significantly increased (*P* < 0.05), whereas Bcl-2 was significantly decreased (*P* < 0.05) in the WB-affected PM muscle, indicating the occurrence of apoptosis mediated by the mitochondrial pathway. Additionally, the expressions of necroptosis-related factors RIP1, RIP3, and MLKL, as well as NF-κB and the pro-inflammatory cytokines TNF-α, IL-1β, and IL-6, were all significantly enhanced (*P* < 0.05) in the WB-affected PM muscle.

**Conclusions:**

The WB myopathy reduces blood supply and induces hypoxia in the PM muscle, which is closely related to the occurrence of PCD including apoptosis, autophagy, and necroptosis within myofibers, and finally leads to abnormal muscle damage and the development of WB in broilers.

## Background

 Chicken is highly favored by consumers due to its unique flavor and comprehensive nutritional content. In response to increasing consumer demand, broilers have been intensively selected for enhanced muscle mass and accelerated growth rate [1]. The rise in broiler chicken production efficiency has led to breast muscle myopathies in broiler chickens, including white striping and wooden breast (WB) [1]. The outer surface of the WB-affected pectoralis major (PM) muscle appears with turbid viscous coating, and small hemorrhages to the muscle fibers, with obvious muscle rigidity and hardness [2]. Such abnormal conditions reduce consumer acceptability and seriously damage chicken quality and nutritional value [1, 3, 4]. Xing et al. [3] reported that a significant number of commercially processed broiler fillets in China are impacted by WB myopathy, with around 30.8% of the poultry population exhibiting moderate to severe WB myopathy. However, the causative factors of WB defects remain unclear.

Petracci et al. [1] suggested that the fast growth rate of modern broilers contributed to the increasing incidence of WB myopathy. Intense selection of broilers based on breast mass and maximum growth rate leads to decreased capillary supply in their PM muscles [4, 5]. The blood vessel density was also reduced in WB-affected PM muscle, suggesting that decreased blood supply and tissue hypoxia may be involved in the development of WB myopathy [6]. At the molecular level, the key pathway for cellular oxygen sensing is the hypoxia-inducible factor-1α (HIF-1α). Under hypoxic conditions, the upregulation of HIF-1α expression promotes the transcription of downstream genes involved in the response to low oxygen levels [7]. Increased expression level of HIF-1α was found in WB-affected PM muscle [8], which was also correlated to expression changes of multiple downstream genes [9]. The above finding suggests a hypoxic state in WB-affected PM muscle.

Hypoxia is one of the important causes of tissue/cell damage [10]. Cells have a limited response to damage, and persistent/severe stimulation can progress to irreversible cell damage [10]. Programmed cell death (PCD) caused by irreversible damage cannot restore normal cell structure and function even after stress relief [10, 11]. Recent research found that WB myopathy was linked to severe damage to the PM muscle, and muscle fibers are unable to fully restore the structure and morphology after being damaged [12]. PCD, which includes autophagy, apoptosis, and necroptosis, refers to the orderly and active death of cells, which is controlled by genes to preserve the stability of the internal environment [11]. Cells may undergo two or three types of cell death when exposed to certain stimuli [11]. Investigating the potential mechanism of hypoxia-induced PCD might be favorable to a more comprehensive understanding of the etiology in WB-affected PM muscle of broiler chickens.

Autophagy is a crucial process for cell survival and stress adaptation. When cells experience energy deprivation or organelle damage due to hypoxia, autophagy is induced to break down damaged cells [11]. A large number of small and large-rimmed vacuoles were found in the PM muscle of birds with WB [13], which was likely caused by the autophagy of myofibers [10]. The vacuoles observed in skeletal muscle are referred to as autophagic vacuoles, and their presence is considered the characteristic morphological hallmark of autophagic vacuolar myopathy [10, 13]. Autophagosomes with double membranes were found in WB-affected PM muscle, enveloping organelles including the small and clear mitochondria [13]. Nichenko et al. [14] pointed out that the appearance of autophagosomes was related to the damage of skeletal muscle cells. Excessive autophagy not only contributes to muscle catabolism and loss but also causes the deterioration of cell function and aggravated cell damage [15]. In addition to autophagy, apoptosis is also a crucial self-destructive process to maintain cellular homeostasis after stress. The purpose of apoptosis is to remove damaged cells from the body with minimal damage to surrounding tissues, which is a response to the disruption of cellular homeostasis caused by stress [10]. Malila et al. [16] reported the occurrence of apoptotic processes in WB by transcriptomic analysis. Greene et al. [8] found that apoptosis was activated in both WB-affected tissues and hypoxic myotube culture. Under hypoxia, mitochondrial pathway that play a key role in apoptosis are activated [17]. Our recent study showed mitochondrial dysfunction in WB-affected PM muscle [12], which is an important manifestation of apoptosis [18, 19]. Cells/tissue damage caused by excessive activation of apoptosis is an important driving force of many diseases and promotes organ damage and dysfunction [18, 20]. Besides, another process of PCD is related to the hypoxic responses, named necroptosis [21]. Necroptosis is characterized by swelling and membrane rupture, and the release of inflammatory cytokines [20]. Zhang et al. [21] reported that hypoxia promotes necroptosis and inflammation while inhibiting necroptosis dramatically reduces the production of pro-inflammatory cytokines under hypoxia conditions. Histological evidence showed that extensive necrosis of existing muscle fibers tissue with infiltration of inflammatory cells in WB-affected PM muscle [2]. Those inflammatory cells were recruited to remove necrotic debris [22]. Consistently, the expression levels of pro-inflammatory factors including tumor necrosis factor-alpha (TNF-α) and interleukin (IL)-1β were increased in WB [23]. Bordini et al. [24] supported a relationship between TNF-induced necroptosis and WB occurrence and progression by a Weighted Gene Co-expression Network Analysis. Necroptosis of muscle cells contributes to accelerated muscle inflammation and subsequent muscle damage [25]. In summary, enhanced hypoxia in the WB-affected PM muscle is highly correlated to the evocation of multiple PCD processes, and is likely a major contributor to the development of this myopathy.

To date, our understanding of the contribution of hypoxia-induced PCD to the formation of WB myopathy remain unclear. Hence, the present study was designed to compare the histological, capillary supply, blood parameters, and the occurrence of PCD in PM muscle between normal and WB myopathic birds to elucidate the underlying mechanisms of hypoxia-mediated PCD in the formation of WB myopathy.

## Methods

### Experimental broiler chickens selection and sample collection

Experimental procedures and bird management were sanctioned by the Institutional Animal Care and Use Committee of Nanjing Agricultural University. The protocol number is SYXK 2021-0014. In this study, broiler chickens (Arbor Acres male) were raised in layered cages and received commercial diets, as well as commercial husbandry routines. Water and feed were provided ad libitum to broiler chickens. A total of 80 broilers aged 42 d were examined for WB myopathy, involving visual observations for posture, and wing contact, as well as bilateral manual palpation for hardness of the PM muscle in a cranio-caudal direction. According to the method proposed by Papah et al. [26], 12 suspected WB-unaffected (easily lift their wings and no detectable hardness of the breast area) and 12 WB-affected live birds (unable to lift their wings sufficiently, and their breast area is markedly firm upon palpation) were selected, and then electrically stunned (alternating current, 400 Hz, 50 V, 5 s each). Blood samples were taken from the brachial vein of live birds, and then the birds were immediately slaughtered via exsanguination. Broiler chickens were necropsied according to the standard reported by Papah et al. [26]. Eight WB-unaffected fillets (showing no detectable increase in firmness of the breast area) and eight WB fillets (exhibiting moderate firmness in a localized area or marked firmness with diffuse coverage of the breast area) were eventually selected through palpation of the PM muscle by three trained personnel. Breast muscles from both sides of the carcass were removed and weighed. The right breast muscles were used for appearance observation and dimension measurement (length, width, and height) were assessed with an electronic caliper as previously described by Baldi et al. [27]. Samples were collected from the superficial layer of the left PM muscles (cranial part), and fixed in paraformaldehyde (4%) for histological observation. The remaining PM muscle tissues were stored at −80 °C for subsequent biochemical assessments.

### Microscopic analysis

The muscle tissues paraffin-embedded blocks were used for histological analysis. The muscle tissue was cut into 6-µm thick cross sections and subjected to hematoxylin and eosin (H&E) staining for histological confirmation of WB myopathy and periodic acid Schiff reaction (PAS) staining to visualize the blood vessels, respectively. Images were acquired by using a light microscope (Axio Scope.A1, Carl Zeiss, Oberkochen, Germany). According to the method of Clark and Velleman [28], the normal breast and WB were performed and ensured through the examination of the microscopic structure, followed by histological evaluations. Muscle fiber diameters and myofiber area were measured using Image-Pro Plus software, version 6.0 (Media Cybernetics, Inc., Rockville, MD, USA).

The measurement of muscle fiber diameter was conducted following the method of Xing et al. [23] with slight modifications. A microscopic field was randomly selected and 10 fibers surrounding an arbitrary fiber in this field were measured. This process was repeated 6 times for each sample and the average was used for the calculations. Moreover, capillary supply, represented as capillary density (capillaries/mm^2^), and a measurement of the ratio of capillaries:muscle fiber, which was determined by examining the number of capillaries relative to myofiber area (measured by mm^2^) or the number of capillaries per myofibers. Eight microscopic field of view of PAS-stained sections were photographed in areas representative of the typical morphology of tissue sections but without large vessels. Each muscle fiber incompletely presented in the field of view was counted as half a fiber and entirely degenerate myofibers with a disrupted endomysium were excluded, and then, the number of microvessels and myofibers was counted manually.

### Blood analysis

Several studies have reported the analysis of blood samples from live birds’ brachial veins [29, 30]. Referring to the method of Lake et al. [29], 1 mL of blood was collected from the brachial wing vein of each bird. The blood was immediately placed into an epoc BGEM test card inserted into epoc Blood Analysis System (Siemens, Tarrytown, NY) to perform rapid blood analysis. Blood chemistry parameters were tested by using BGEM test cartridges, including pH, partial pressure of carbon dioxide (pCO_2_), total carbon dioxide (TCO_2_), bicarbonate (HCO_3_^−^), partial pressure of oxygen (pO_2_) and saturation oxygen (sO_2_), as well as sodium (Na^+^), potassium (K^+^) and ionized calcium (iCa). Finally, all data was downloaded from the analyzer and subjected to statistical analysis.

### Detection of caspase 9 and caspase 3 activation degree

As per manufacturer’s instructions, the activation degree of caspase-9 (#G018-1-2) and caspase 3 (#G015-1-2) were measured by using the commercial kits (Nanjing Jiancheng Bioengineering Institute, Nanjing, China).

### Apoptotic nuclei analysis

The detection of apoptotic cell nuclei was performed by using a terminal deoxynucleotidyl transferase (TdT)-mediated dUTP nick-end labeling (TUNEL) kit (Vazyme Biotech Co., Ltd., Nanjing, China), according to the manufacturer’s instructions. Then a fluorescence microscope (Axio Scope.A1, Carl Zeiss, Oberkochen, Germany) is used to observe TUNEL-positive nuclei. Following the method of Xing et al. [31], four fields were randomly selected for analysis using the Image Pro Plus software, version 6.0 (Media Cybernetics, Inc., Rockville, MD, USA), for apoptotic nuclei evaluation. Finally, the cell apoptotic index was calculated as the percentage of the total number of nuclei.

### Determination of ATP, AMP, and ADP content

High-performance liquid chromatography (HPLC) was used to determine the contents of ATP, AMP, and ADP. After being removed from the −80 °C refrigerator, a 0.3 g sample was homogenized for 1 min in 2 mL of cold 5% HClO_4_ solution, and then placed for 15 min at 4 °C for extraction. Then, the homogenate was centrifuged for 10 min at 4 °C at 15,000 × *g*. 850 µL supernatant was collected, adjusted the pH to 6.5 using a 1.03 mol/L KOH solution, and then centrifuged again at 15,000 × *g* for 10 min at 4 °C. The supernatant was filtrated using a 0.45 μm filter to further the HPLC test (Waters-2695 Alliance). The test settings were as follows: sample loading 10 µL, column temperature 30 °C, flow rate 1 mL/min, UV detection wavelength 245 nm, and detection time 20 min. Mobile phase A was chromatographically pure methanol. Mobile phase B was sodium dihydrogen phosphate buffer, including 2.5 mmol/L tetrabutylammonium hydrogen sulfate, 0.04 mmol/L potassium dihydrogen phosphate, and 0.06 mmol/L dipotassium hydrogen phosphate. The pH value of the buffer was adjusted to 6.5. The proportions of mobile phases A and B were 13.5% and 86.5%. Results were calculated according to an established standard curve. The standard samples of 5´-ATP disodium salt, 5´-ADP sodium salt, and 5´-AMP sodium salt were purchased from Sigma-Aldrich Company (St. Louis, MO, USA).

### RNA purification and real-time quantitative PCR analysis

RNAiso plus reagent (Takara Biotechnology Co., Ltd., Dalian, China) was used to isolate total mRNA from PM muscles. The mRNA was reversely transcribed into cDNA using a cDNA synthesis commercial kit (Takara Biotechnology Co., Ltd.). SYBR Premix Ex Tap (Vazyme Biotechnology Co., Ltd., Nanjing, China) was used for real-time quantitative polymerase chain reaction (qPCR) on an ABI PRISM 7500 (Applied Biosystems, Foster City, CA, USA). Table S1 (additional file 1) shows the primer sequences used for qPCR. In our present study, the target genes that were tested including light chain 3 (*LC3*) I, *LC3 II*, *HIF-1α*, Bcl2/Adenovirus E1B 19 kDa interacting protein 3 (*BNIP3*), *Beclin1*, adenosine monophosphate-activated protein kinase alpha (*AMPKα*) 2, UNC-51-like kinase 1 (*ULK1*), *caspase9*, *caspase3*, Bcl-2-associated X protein (*Bax*), B cell lymphoma 2 (*Bcl-2*), cytochrome c (*Cytc*), receptor-interacting protein (RIP) 1, *RIP3*, mixed lineage kinase domain-like protein (*MLKL*), nuclear factor kappa-light-chain enhancer of activated B cells (*NF-κB*), *TNF-α*, *IL-1β*, *IL-6*. The 2^−∆∆Ct^ method with Tubulin as the internal reference gene was used to calculate the relative mRNA expression.

### Total protein extraction and western blot analysis

RIPA lysis buffer (Beyotime Biotechnology, Jiangsu, China), which contains phosphatase and protease inhibitors, was added to frozen PM muscle samples and then homogenized. The homogenate was centrifuged at 4 °C for 20 min at 12,000 × *g*, and the supernatant was collected. The BCA kit was used to detect the protein concentration. Equal amounts of total protein were electrophoresed on SDS-PAGE (10% or 12.5%) and transferred to a polyvinylidene fluoride membrane (Millipore; Billerica, MA, USA). At room temperature, bovine serum albumin (5%) was used to block the membranes for 1 h. Next, the membranes were incubated in primary antibodies against LC3 (ProteinTech, Wuhan, China), Beclin1, AMPKα, p-AMPKα (Cell Signaling Technology, Danvers, MA, USA), HIF-1α, Caspase3 (NOVUS, Littleton, USA), NF-κB, Cytc (Servicebio Biological Technology, Wuhan, China), Bax, p-NF-κB (Wanleibio, Shenyang, China), ULK1, p-ULK1, RIP1, p-RIP1, RIP3 (Abclonal, Wuhan, China), p-RIP3 (AiFang biological, Changsha, China), MLKL (Affinity Biosciences, Bejing, China), p-MLKL (Abcam, Cambridge, UK) and Tubulin (Beyotime, Shanghai, China) overnight at 4 °C with gentle shaking. At last, at room temperature, the membranes were incubated by using the corresponding secondary antibodies (Cell Signaling Technology, Danvers, MA, USA) for 1 h. The protein bands were treated with an ECL chemiluminescence detection kit (Vazyme Biotechnology Co., Ltd., Nanjing, China) and then visualized with the ChemiDoc™ Imaging System (BIO-RAD, Singapore). Quantity One software (Bio-Rad) was used to quantify the density of bands and the relative protein expression was calculated by Image J software (version 1.44p, National Institutes of Health, Bethesda, MD, USA) with Tubulin as a normalized control. The data in the CON group were normalized to 1, and the data in the WB group were calculated as the fold of the CON group.

### Immunohistochemistry

The muscle tissues’ paraffin-embedded blocks were used for immunohistochemistry (IHC). Dewaxing in xylene and ethanol; preheating sodium citric acid (pH = 6.0) antigen retrieval solution at high temperature for 10 min, the tissue section is boiled in the solution for 30 min and 3% H_2_O_2_ for 30 min, then 10% BSA for 30 min. p-RIP3 antibody (AiFang biological, Changsha, China) was diluted with 10% BSA at a ratio of 1:200. The slides were incubated with p-RIP3 overnight at 4 °C and then DAB solution for 4 min and hematoxylin counterstain for 1 min. Hydrochloric acid ethanol differentiation for 1–2 s, and then dehydrated with ethanol for 2 min. After drying at room temperature, neutral gum was added to mount the slide. Fields of view were randomly selected under the microscope.

### Statistical analysis

SPSS Statistics (SPSS, Inc. Chicago, IL, USA) was used for data analysis and Student’s *t*-tests were used to compare the differences between the groups of CON and WB (*n* = 8), with the exception of western blot analysis (*n* = 6). Data were reported as mean ± standard error (SE). A trend was considered at 0.05 < *P* < 0.1, and significance was indicated at *P* ≤ 0.05.

## Results

### Breast muscle characteristics and morphology

**Fig. 1:**
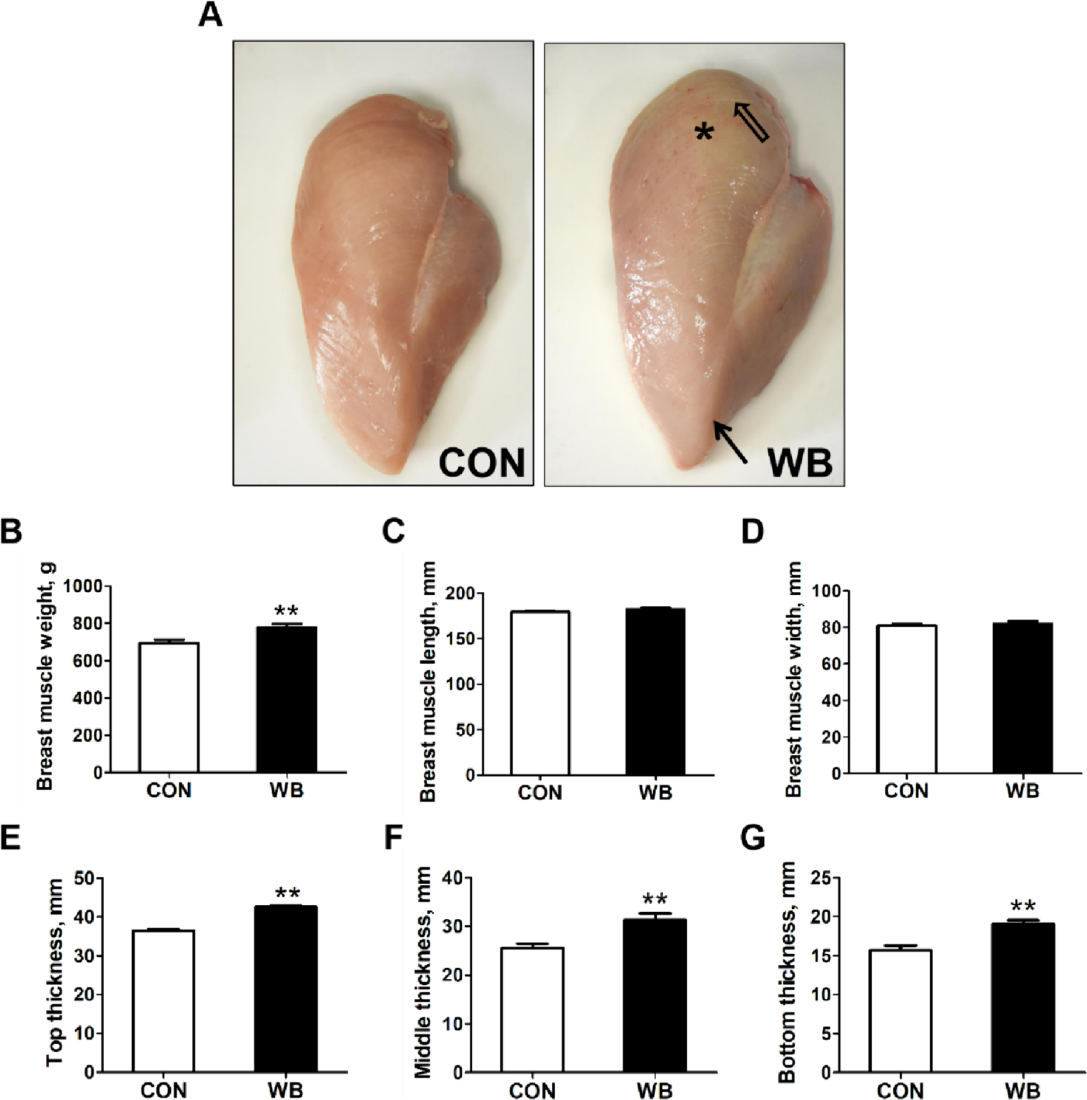
Breast muscle appearance, weight and dimension of raw chicken fillets of normal breast (CON) meat and wooden breast (WB). **A** Appearance of breast muscle. In WB-affected breast muscle, the surface was covered with moderately viscous material, as well as white striping (open arrow) and small hemorrhages (asterisk) and pale ridge-like bulges in muscle bottom (arrow). **B** Breast muscle weight of birds. **C** Length of breast muscle. **D** Width of breast muscle. **E** Top thickness of breast muscle. **F** Middle thickness of breast muscle. **G** Bottom thickness of breast muscle. Data are expressed as the mean ± SE (*n* = 8 per group). SE, standard error. ^**^P ≤ 0.01

Moderately viscous material, as well as small hemorrhages and white striping, was found in the PM muscle of the WB group (Fig. 1A). Moreover, the weight was increased, and the thickness (top, middle, and bottom) of the WB were thicker than those of the CON (*P* < 0.01, Fig. 1B, E–G).

**Fig. 2:**
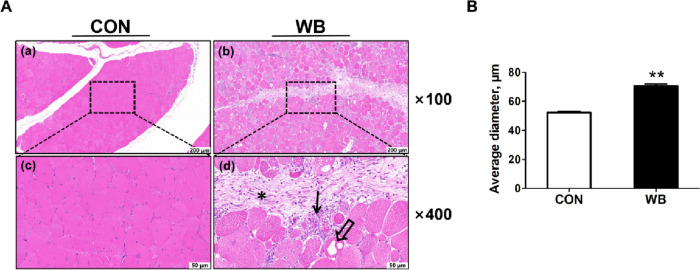
Histopathological observation and results of muscle fiber average diameter of normal breast (CON) meat and wooden breast (WB). **A** Representative images of hematoxylin and eosin staining of PM muscle at different magnifications. Vacuolar degeneration (open arrow); Degenerating fibers (arrow) are often surrounded by inflammatory cell infiltration and abundant collagen-rich connective tissue (asterisk). **B** Average diameters in PM muscle. Figures (a) and (b) are magnified at 100×, with a scale bar at 200 μm; figures (c) and (d) are magnified at 400×, with a scale bar at 50 μm. Data are expressed as the mean ± SE (*n* = 8 per group). SE, standard error. ^**^P ≤ 0.01

H&E staining showed that muscle fibers from the CON group were polygonal and tightly arranged with no degenerative fibers. On the contrary, the WB muscle fibers were round and loosely arranged. An accumulation of inflammatory cells and interstitial connective tissue was observed in WB-affected PM muscle (Fig. 2A). The mean muscle fiber diameter in WB was considerably higher than that of CON (*P* < 0.01; Fig. 2B).

### Capillary supply and blood analysis

As shown in Table 1, the number of capillaries per fiber was notably lower in the WB than in the CON (*P *< 0.01). Moreover, the blood pO_2_, sO_2_, and pH of WB-affected birds were substantially lower than those of unaffected birds (*P *< 0.01). pCO_2_, TCO_2_, and K^+^ values in WB-affected birds were noticeably greater than in normal birds (*P *< 0.05).

**Table 1:** Capillary supply in pectoralis major muscle and blood parameters of normal breast (CON) and wooden breast (WB) birds

Items	Treatment		SEM	*P*-value
	CON	WB		
Capillary supply				
capillaries/fiber	1.01	0.81**	0.01	<0.01
capillaries/mm^2^	306.90	288.94	9.81	0.219
Blood parameters				
pO_2_, mmHg	48.51	39.65**	1.05	<0.01
sO_2_, %	84.23	70.25**	1.86	<0.01
pCO_2_, mmHg	41.99	47.30*	1.42	0.027
TCO_2_, mmol/L	26.36	29.15**	0.58	0.004
HCO_3_^−^, mmol/L	24.29	25.28	0.57	0.257
pH	7.39	7.29**	0.02	0.005
Na^+^, mmol/L	148.75	149.50	0.62	0.409
K^+^, mmol/L	6.04	6.54**	0.10	0.006
iCa, mmol/L	1.60	1.57	0.01	0.189

*pO*_2_ Partial pressure of oxygen, *sO*_2_ Oxygen saturation, *pCO*_2_ Partial pressure of carbon dioxide, *TCO*_2_ Total carbon dioxide, *HCO*_*3*_^−^ Bicarbonate, *Na*^+^ Sodium, *K*^+^ Potassium, *iCa* Ionized calcium, *SEM* Standard error of mean. Data are expressed as the mean and SEM (*n* = 8)

### Expression of autophagy-related genes and proteins in the PM muscle

**Fig. 3:**
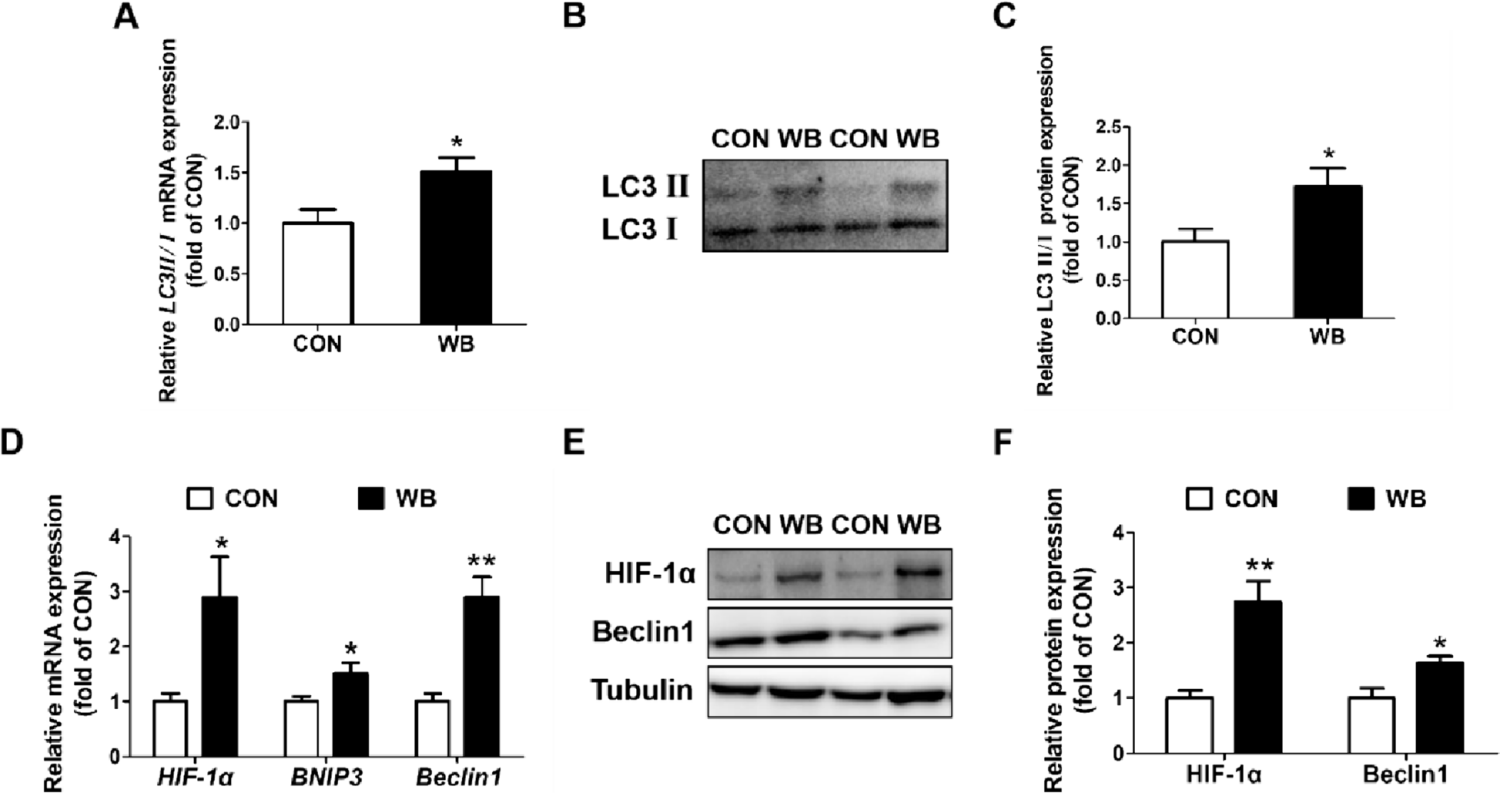
Expression of autophagy-related genes and proteins in pectoralis major muscle of CON and WB birds. **A** Relative mRNA expression of light chain 3 II/I (*LC3 II/I*). **B** Representative western blotting images of LC3 II and LC3I. **C** Relative protein expression of LC3 II/I. **D** Relative mRNA expressions of hypoxia-inducible factor 1α (*HIF-1α*), recombinant Bcl2/Adenovirus E1B 19 kDa Interacting Protein 3 *(BNIP3)* and *Beclin1*. **E** Representative western blotting images of HIF-1α and Beclin1. **F** Relative protein expressions of HIF-1α and Beclin1. Data are expressed as the mean ± SE for mRNA expressions (*n* = 8 per group) and protein expressions (*n* = 6 per group). ^**^P ≤ 0.01 and ^*^0.01 < *P* ≤ 0.05

**Fig. 4:**
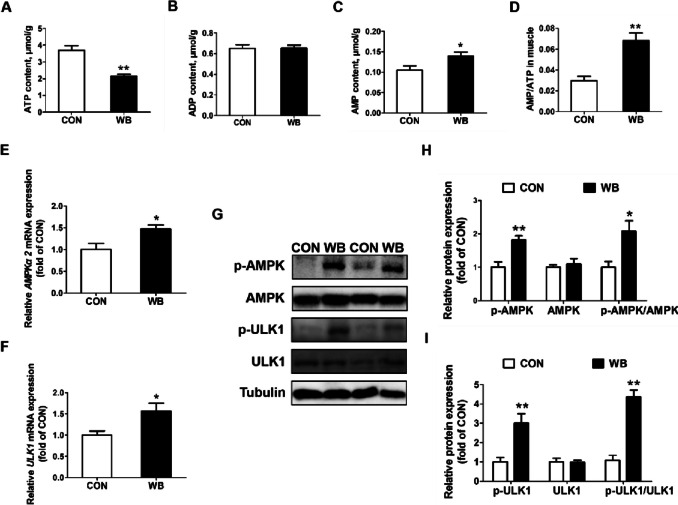
Energy status and AMPK/ULK1 pathway in pectoralis major muscle of CON and WB birds. **A** ATP content. **B** ADP content. **C** AMP content. **D** AMP/ATP ratio. **E**–**F** Relative mRNA expressions of adenosine monophosphate-activated protein kinase alpha 2 (*AMPKα2*) and UNC-51-like kinase 1 (*ULK1*). **G** Representative western blotting images of p-AMPK, AMPK, p-ULK1 and ULK1. **H** Relative protein expressions of p-AMPK and AMPK. **I** Relative protein expressions of p-ULK1 and ULK1. Data are expressed as the mean ± SE for energy status and mRNA expressions (*n* = 8 per group) and protein expressions (*n* = 6 per group). ^**^P ≤ 0.01 and ^*^0.01 < *P* ≤ 0.05

The ratios of LC3 II to LC3 I at the mRNA and protein levels were considerably higher in the WB group than in the CON group (*P* < 0.05; Fig. 3A–C). In contrast to CON, WB had higher mRNA expression levels of *HIF-1α*, *BNIP3*, and *Beclin1* along with the protein levels of HIF-1α and Beclin1 were also enhanced in the WB group (*P* < 0.05; Fig. 3D–F). In addition, WB also decreased the ATP content (*P* < 0.01; Fig. 4A), but increased the AMP content (*P* < 0.05; Fig. 4C) and the AMP/ATP ratio (*P* < 0.01; Fig. 4D). Moreover, elevated mRNA expressions of *ULK1* and *AMPKα2* was detected in the WB group (*P* < 0.05; Fig. 4E and F). Consistently, the protein expressions of p-AMPK and p-ULK1, the ratio of p-AMPK to AMPK, and the ratio of p-ULK1 to ULK1 were all enhanced in PM muscle with WB myopathy (*P* < 0.05; Fig. 4G–I).

### Apoptotic index and expression of apoptosis-related genes and proteins in the PM muscle

**Fig. 5:**
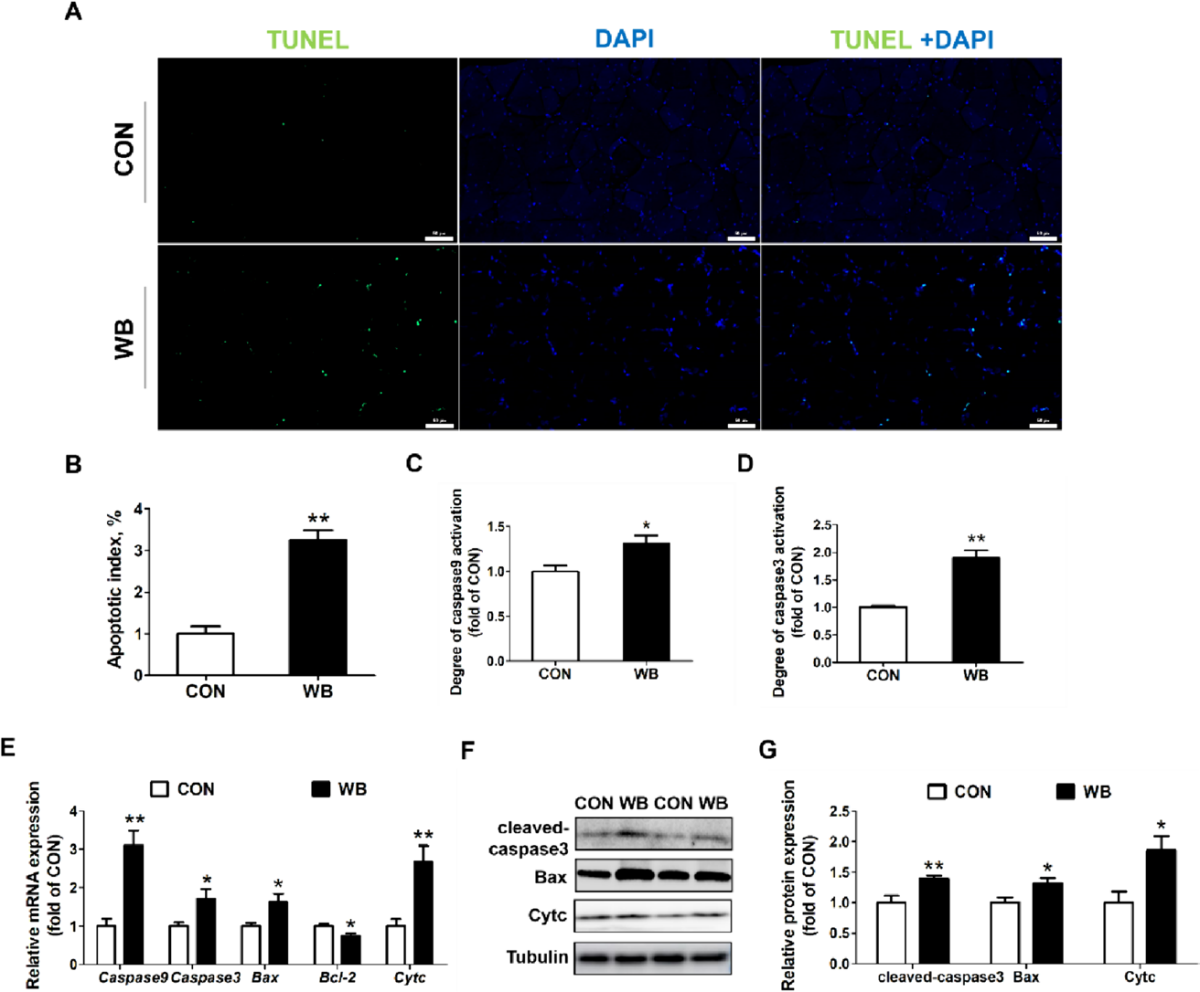
Apoptotic status and apoptosis-related mediators in the pectoralis major (PM) muscle of CON and WB birds. **A** Representative images of terminal deoxynucleotidyl transferase-mediated dUTP nick-end labeling (TUNEL) staining of PM muscle. **B** Relative percentage of muscle cell apoptosis. **C **and **D** Relative activities of caspase 9 and caspase3. **E** Relative mRNA expressions of *caspase 9*, *caspase3*, Bcl-2-associated X protein (*Bax*), B cell lymphoma (*Bcl*)-2 and cytochrome c (*Cytc*). **F** Representative western blotting images of cleaved-caspase 3, Bax and Cytc. **G** Relative protein expressions of cleaved-caspase 3, Bax and Cytc. All scale bars represent a length of 50 μm. Data are expressed as the mean ± SE for activities of caspase 9 and caspase 3 (*n* = 8 per group), mRNA expressions (*n* = 8 per group) and protein expressions (*n* = 6 per group). ^**^P ≤ 0.01 and ^*^0.01 < *P* ≤ 0.05

TUNEL labeling was used to identify the apoptotic muscle cell nucleus. As shown in Fig. 5A, the apoptotic index was significantly higher in WB-affected muscles compared to the CON group (*P* < 0.01; Fig. 5B). The activities of caspase 9 (*P* < 0.01; Fig. 5C) and caspase 3 (*P* < 0.05; Fig. 5D) were also elevated in the WB group along with enhanced mRNA expressions of *caspase 9* and *caspase 3*. In addition, pro-apoptotic factors including *Bax* and *Cytc* were upregulated in the WB group while the anti-apoptotic regulator of *Bcl-2* was decreased (*P* < 0.05; Fig. 5E). At protein levels, WB also enhanced expressions of cleaved-caspase 3, Bax, and Cytc (*P* < 0.05; Fig. 5F and G).

### Expression of necroptosis-related genes and proteins in the PM muscle

**Fig. 6:**
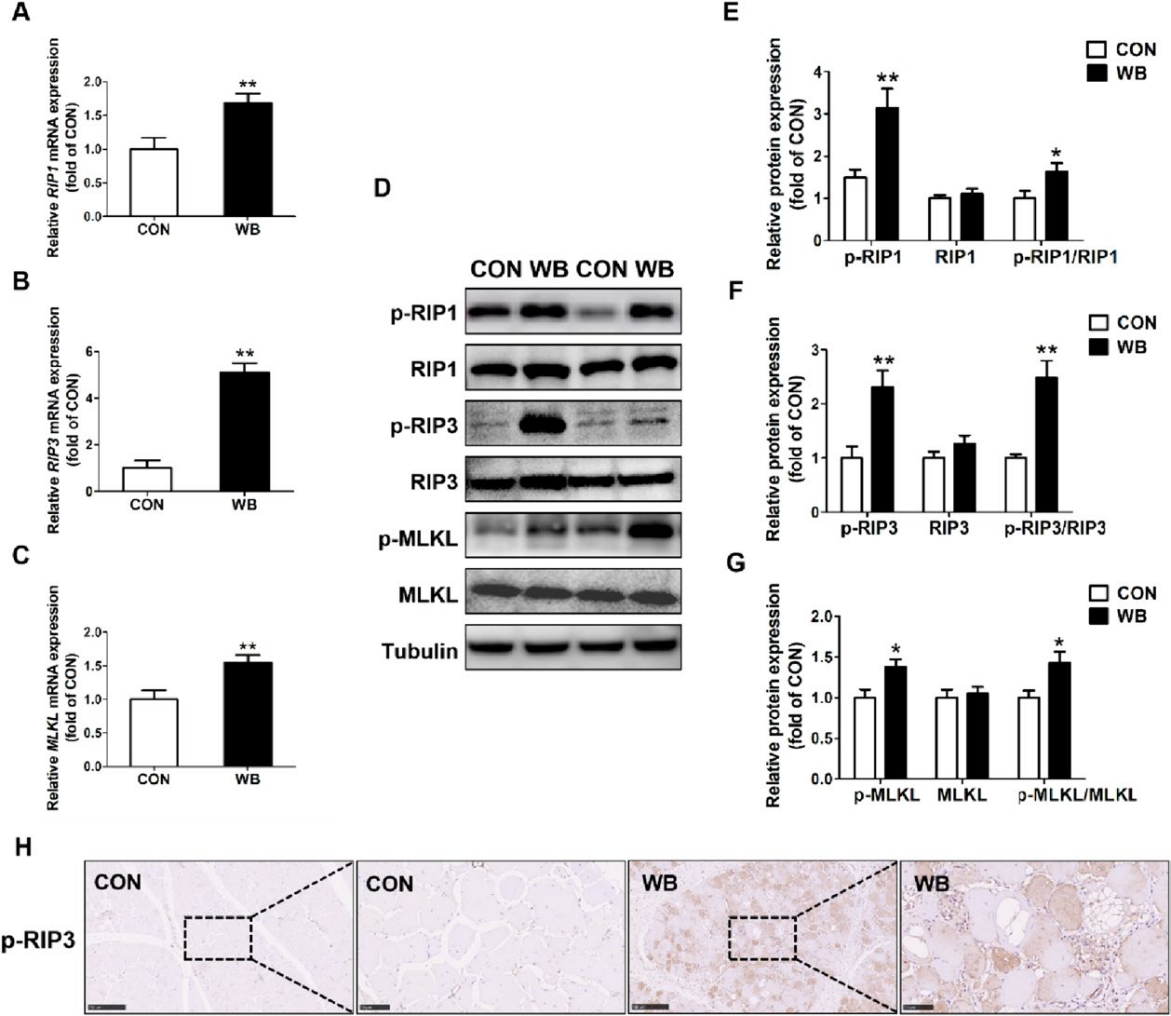
Necroptosis-related genes in pectoralis major muscle of CON and WB birds. **A**–**C** Relative mRNA expressions of receptor-interacting protein (*RIP*)1, *RIP3*, mixed lineage kinase domain-like protein (*MLKL*). **D** Representative western blotting images of p-RIP1, RIP1, p-RIP3, RIP3, p-MLKL and MLKL. **E**–**G** Relative protein expressions of p-RIP1, RIP1, p-RIP3, RIP3, p-MLKL and MLKL. **H** Immunohistochemistry detected the distribution of p-RIP3 in the PM muscle. Data are expressed as the mean ± SE for mRNA expressions (*n* = 8 per group) and protein expressions (*n* = 6 per group). ^**^P ≤ 0.01 and ^*^0.01 < *P* ≤ 0.05

**Fig. 7:**
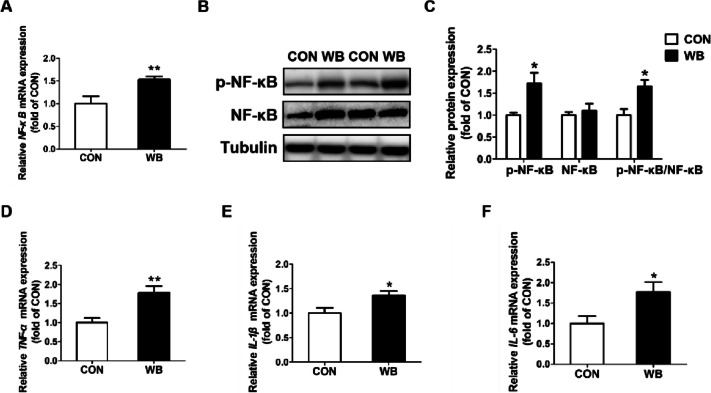
NF-κB and its downstream targets in pectoralis major muscle of CON and WB birds. **A** Relative mRNA expressions of nuclear factor kappa-light-chainenhancer of activated B cells (*NF-κB*). **B** Representative western blotting images of p-NF-κB and NF-κB. **C** Relative protein expressions of p-NF-κB and NF-κB. **D**–**F** Relative mRNA expressions of tumor necrosis factor-alpha (*TNF-α*), interleukin (*IL*)*-1β* and *IL-6*. Data are expressed as the mean ± SE for mRNA expressions (*n* = 8 per group) and protein expressions (*n* = 6 per group). ^**^P ≤ 0.01 and ^*^0.01 < *P* ≤ 0.05

The mRNA and protein expression of necroptosis-related factors were determined. The mRNA expressions of *RIP1*, *RIP3*, and *MLKL* were significantly higher in the WB group compared to the CON group (*P* < 0.01; Fig. 6A–C). In comparison to the CON birds, WB broiler chickens consistently showed significantly up-regulated protein expressions of p-RIP1, p-RIP3, and p-MLKL in the PM muscle, as well as the ratios of p-RIP1/RIP1, p-RIP3/RIP3, and p-MLKL/MLKL (*P* < 0.05, Fig. 6D–G). IHC assay showed that p-RIP3 was mainly distributed in the cytoplasm in the PM muscle of broiler chickens with WB myopathy (Fig. 6H). Furthermore, increased mRNA expression of *NF-κB* and protein contents of p-NF-κB, as well as the ratio of p-NF-κB to NF-κB were found in WB-affected PM muscle, suggesting activated NF-κB inflammatory signaling (*P* < 0.05; Fig. 7A–C). Consistently, the mRNA expressions of NF-κB signaling downstream targets, including *TNF-α*, *IL-1β*, and *IL-6*, were significantly increased in the WB group compared to the CON group (*P* < 0.01; Fig. 7D–F).

## Discussion

The occurrence of WB myopathy seriously affects the appearance of chicken breasts, and reduces consumers’ desire to buy [1]. In this study, the WB-affected PM muscles exhibited remarkably hard areas, with small hemorrhages and viscous fluid on the outer surface, which was consistent with the report of Sihvo et al. [2]. A meta-analysis identified that high growth (measured as daily weight gain and slaughter weight) could be a factor that increases the risk of occurrence of this myopathy [32]. The increased incidence of WB is known to be associated with increased breast muscle yield and thickness [1, 27], which was also confirmed by this study. Increased muscle growth is the result of the hypertrophic growth of muscle fiber [5]. Xing et al. [23] found that the fiber diameter of the WB-affected PM muscle increases with the weight of the breast fillet. According to the current findings, WB birds had a markedly higher average muscle fiber diameter, suggesting hypertrophy of muscle fibers in WB-affected PM muscle [4, 5]. Myofiber hypertrophy reduces the available space for capillaries, leading to tissue hypoxia and ultimately increasing the potential for myofiber damage [4, 5]. Histological examination clearly showed myofiber destruction in the PM muscle of birds with WB, with clear necrotic myopathic lesions, vacuolation, inflammatory cell infiltration, and connective tissue deposition. Additionally, it was reported that the muscles of birds affected by WB had separated and disintegrated myofibrils, indicating that the skeletal muscle’s structure had also been compromised [12].

Overgrowth of breast muscle is achieved by myofiber hypertrophy, which impairs the capillary supply to the muscle by reducing the amount of perimysial and endomysial accessible space for capillaries [4–6]. This study also found a significant decrease in the number of capillaries per fiber and a decreased tendency in the number of capillaries/mm^2^ in the WB group, which is similar to the report by Sihvo et al. [6], indicating a reduction in capillary supply. Fast-growing broilers have a high O_2_ demand [33]. Abnormal changes in the levels of O_2_ and CO_2_ present a severe threat to cell survival. After examining the venous blood of 35-day-old male broilers, Livingston et al. [30] discovered substantial correlations between the WB severity and both the increase in total CO_2_ and the decrease in pO_2_. Similarly, in this study, WB-affected birds exhibited significantly lower sO_2_ and pO_2_, in addition to having a substantially higher pCO_2_ than the CON. As discussed by Lake et al. [29], the altered venous blood gas values indicated inadequate respiratory gas exchange in the WB-affected birds. The imbalance of O_2_ and CO_2_ levels in the blood changes the pH of the blood [34]. In the present study, the WB-affected birds also showed a significant decline in pH and higher K^+^ levels caused by high blood CO_2_, indicating blood gas disturbances [29, 34]. Decreased supply of O_2_ induces hypoxia and HIF-1α is the most crucial responsor for hypoxic conditions. Previous studies have reported the enhanced expression of HIF-1α and its associated downstream genes in the WB-affected birds [8, 16]. Greene et al. [35] suggested that WB-affected broiler chickens were associated with dysregulated oxygen homeostasis and hypoxia status. Emami et al. [36] found that hypoxia exacerbated WB incidence in broilers. Prolonged exposure to extreme hypoxia is harmful to muscle structure and typically causes muscle damage [37]. Collectively, the aforementioned findings suggested the occurrence of hypoxia in WB-affected PM muscle, which was caused by the reduction of capillary supply and the abnormality of gas exchanges, which may lead to muscle injury. However, the contribution of hypoxia to myofiber damage and the development of WB myopathy remains unclear.

Autophagy is stimulated rapidly after hypoxia and leads to autophagic cell death under severe hypoxic conditions [10, 11]. Excessive autophagy is detrimental to muscle homeostasis and leads to the occurrence of various types of skeletal muscle disease including WB myopathy in broilers [15]. Muscle fiber vacuolation was found in WB-affected PM muscle in this study, which was likely caused by autophagy similar to the common role of autophagy in other chronic muscle degenerative disorders [10, 13]. Autophagosome formation and fusion is a necessary biological process in autophagy and is mainly regulated by microtubule-associated protein LC3 [11]. There are two types of LC3 proteins, LC3-I and LC3-II. LC3-II incorporates into the outer and inner membranes of the autophagosome and is considered a common indicator of occurrence of autophagy [11]. Herein, our results showed that the mRNA and protein ratio of LC3 II to LC3 I were elevated in WB-affected PM muscle. Moreover, hypoxia induced autophagy by enhancing the expression of HIF-1α and its downstream target of BNIP3 [18]. According to a previous study, BNIP3 resulted in the loss of skeletal muscle mass and was considered a possible target for treatment in autophagy-related disorders such as muscle wasting diseases [37]. Beclin1 is a downstream target of BNIP3 and promotes autophagosome formation [18]. Chen et al. [38] found that CoCl_2_ (a hypoxia-mimetic agent), induced autophagy through HIF-1α/BNIP3/Beclin1 signaling pathway. Consistently, elevated expressions of HIF-1α, BNIP3, and Beclin1 were found in WB-affected PM muscle in this study, which initiated the subsequent autophagic process. Furthermore, low-energy status also initiates autophagy [11]. Wang et al. [39] found that there was a negative energy balance in WB-affected PM muscle through an integrative analysis of transcriptomic and metabolomic. Our previous study reported the dysfunction of mitochondria in the WB-affected PM muscle, which might cause a shortage of energy supply [12]. Low oxygen stress causes a reduction in mitochondrial oxidative phosphorylation, which raises the ratio of adenosine monophosphate to adenosine triphosphate (AMP/ATP) and decreases ATP production [10, 11]. According to our results, there was a decrease in ATP level but an increase in the AMP/ATP ratio, which is thought to be a key route for triggering AMPK activation in a hypoxic environment [11]. AMPK is a potent autophagy activator and is required for cellular growth and energy homeostasis. Phosphorylated AMPK interacts with ULK1, triggering autophagosome nucleation and elongation [11]. The AMPK/ULK1 pathway was also involved in the autophagy of C2C12 myotubes under hypoxic conditions [7]. Consistently, this study showed that AMPK induced autophagy in WB-affected PM muscle through the phosphorylation of ULK1. Autophagy is a consistent response to skeletal muscle damage [15]. Overall, our results showed that hypoxia might initiate autophagy in WB-affected PM muscle by directly activating the HIF-1α/BNIP3/Beclin1 pathway or through the AMPK/ULK1 pathway caused by low-energy status.

Hypoxia can also induce apoptosis, which is a process to eliminate unwanted cells [10]. Apoptotic signaling in skeletal muscle is typically associated with muscle dysfunction [18]. The TUNEL assay showed a marked increase in the number of muscle nuclei undergoing apoptosis in WB. Apoptosis induced by hypoxia is first manifested as mitochondrial dysfunction, especially the decrease of mitochondrial membrane potential (ΔΨm), which is a crucial apoptotic signature [18, 19]. Our recent study showed mitochondrial dysfunction in WB-affected PM muscle [12]. Weinmann et al. [17] reported that hypoxia-induced apoptosis mainly relies on mitochondrial pathways. The pro-apoptotic (Bax) and anti-apoptotic B-cell lymphoma-2 (Bcl-2) family members play major roles in mitochondira-related apoptosis. Bax oligomerizes at the mitochondrial outer membrane to mediate its permeabilization and promote the release of apoptotic initiating factors such as Cytc and then activate the caspase cascade [11]. Bcl-2 protein can inhibit the effects of Bax. In this study, it was suggested that the mitochondria-mediated apoptosis occurred in the PM muscle apoptosis of WB-affected birds which was driven by the increased expression of Bax, the decreased levels of Bcl-2, and the release of Cytc. The apoptotic process is carried out through the caspase family, caspases 3 and 9 are critical mediators of mitochondrial pathway mediated apoptosis, and caspase 3 activation is dependent on mitochondrial Cytc release and caspase 9 function [11]. Caspase 9 and 3 were upregulated in WB-affected PM muscle in this study. Greene et al. [8] reported that hypoxia induced the increased levels of caspase 9 and caspase 3 in chicken primary muscle cell culture, as well as the increased expression of caspase 3 in WB-affected tissues. It is worth noting that apoptosis must be kept at an equilibrium, too much can cause excessive tissue destruction [10, 18]. Shi et al. [20] reported that apoptosis contributed to tissue damage following spinal cord damage. Overall, the above results indicated that hypoxia might mediate apoptosis in WB-affected PM muscle by the mitochondria-mediated caspase activation pathway.

Necrosis is a significant pathological feature of WB myopathy in broiler chickens. Huang et al. [40] reported that necrotic cell death is triggered by hypoxia through the RIP signaling pathway. Necroptosis is mainly mediated by RIP1 and RIP3. RIP1 combines with RIP3 to form a necrotic complex, which induces the phosphorylation of its substrate MLKL [21]. This leads to the lysis of the membrane, causing uncontrollable release of intracellular material. Results of this study showed that the expressions and phosphorylation of RIP1, RIP3, and MLKL were upregulated in WB, indicating that mechanisms underlying WB occurrence may trigger necroptosis process. Moreover, the IHC results showed that p-RIP3 was predominantly present in the cytoplasm of WB-affected PM muscle. In addition, some positive signals were also detected in myofibers that suffered severe damage with inflammatory cell infiltration. Zhang et al. [21] reported prolonged hypoxia-induced necroptosis and inflammation by activating RIP1 kinase and raising the phosphorylation level of RIP3 and MLKL. Inflammatory response is believed to be the central factor in the pathogenesis of necroptosis-associated diseases [25]. Pediatric patients with inflammatory bowel disease have elevated levels of RIP3 and MLKL in the inflammatory tissue, demonstrating a strong correlation between necroptosis and inflammatory disease [41]. Hypoxia is a common feature of many inflammatory diseases [21]. NF-κB is activated under hypoxic conditions, which plays a crucial role in regulating the immune response and is a central mediator of pro-inflammatory genes [42]. Culver et al. [43] found that NF-κB was activated when cells were exposed to different levels of hypoxia. Hypoxia-induced NF-κB leads to the activation of pro-inflammatory cytokines including TNF-α, IL-1β, and IL-6 [42]. In our present study, NF-κB signaling was activated together with mRNA expressions of *TNF-α*, *IL-1β*, and *IL-6*, which are identified as primary factors in muscle injury and inflammation [22, 25, 42]. TNF-α activation initiates the classic necroptosis pathway by successively activating the RIP1, RIPK3, and MLKL [44]. Therefore, we speculated that the activated NF-κB signaling and inflammatory response by hypoxia promote necroptosis, and muscle damage in WB myopathy. Moreover, the RIP1/RIP3 necrosome complex induces the production of NF-κΒ-dependent cytokines in necroptotic cells [44], which may exacerbate necroptosis and trigger an uncontrolled inflammatory cascade, resulting in severe tissue damage [45]. Overall, the occurrence of necroptosis in WB myopathy might be related to the activation of NF-κΒ signaling caused by hypoxia, which promotes the release of pro-inflammatory factors and initiates a ‘vicious cycle’ of necroptosis and immune disorders, aggravating muscle damage in turn.

## Conclusions

In conclusion, this study investigated the possible mechanism of hypoxia on the damage of myofibers through three types of PCD in broilers with WB. The decrease of capillary supply in the PM muscle and the inadequate respiratory gas exchange suggested hypoxia while the increased expression of HIF-1α and hypoxia-response genes confirmed that hypoxia state in the WB-affected PM muscle. Three types of PCD were evaluated to show the responses of myofibers to hypoxic conditions in the WB-affected PM muscle. Hypoxia may mediate autophagy in WB-affected PM muscle by activating both HIF-1α/BNIP3/Beclin1 pathway and AMPK/ULK1 pathway. Additionally, hypoxia may also mediate apoptosis and necroptosis respectively through the activation of the caspase apoptotic pathway and NF-κΒ signaling. In general, our results shed light on a possible mechanism by which hypoxia mediates PCD and leads to injury to the PM muscle in the occurrence and progression of WB myopathy. The collected results enhance the understanding of the pathophysiological processes of WB and may provide regulatory targets for coping with this myopathy.

## Data Availability

All data generated during this study are available from the corresponding authors on reasonable request.

## Abbreviations

AMPKα2: Adenosine monophosphate -activated protein kinase alpha 2
Bax: Bcl-2-associated X protein
Bcl-2: B cell lymphoma-2
BNIP3: Bcl2/Adenovirus E1B 19kDa interacting protein 3
Cytc: Cytochrome c
H&E: Hematoxylin and eosin
HCO3^−^: Bicarbonate
HIF-1α: Hypoxia-inducible factor-1α
HPLC: High-performance liquid chromatography
iCa: Ionized calcium
IL-1β: Interleukin-1β
IL-6: Interleukin-6
K^+^: Potassium
LC3: Light chain 3
MLKL: Mixed lineage kinase domain-like protein
Na^+^: Sodium
NF-κB: Nuclear factor kappa-light-chain enhancer of activated B cells
PAS: Periodic acid Schiff reaction
PCD: Programmed cell death
pCO_2_: Partial pressure of carbon dioxide
PM: Pectoralis major
pO_2_: Partial pressure of oxygen
qPCR: Quantitative polymerase chain reaction
RIP1: Receptor-interacting protein 1
RIP3: Receptor-interacting protein 3
sO_2_: Oxygen saturation
TCO_2_: Total carbon dioxide
TNF-α: Tumor necrosis factor-alpha
TUNEL: Terminal deoxynucleotidyl transferase (TdT)-mediated dUTP nick-end labeling
ULK1: UNC-51-like kinase 1
WB: Wooden breast
